# Non‐coding RNAs in malaria infection

**DOI:** 10.1002/wrna.1697

**Published:** 2021-10-14

**Authors:** Valeria Lodde, Matteo Floris, Maria Rosaria Muroni, Francesco Cucca, Maria Laura Idda

**Affiliations:** ^1^ Department of Biomedical Sciences University of Sassari Sassari Italy; ^2^ Department of Medical, Surgical, and Experimental Sciences University of Sassari Sassari Italy; ^3^ Institute for Genetic and Biomedical Research (IRGB), National Research Council (CNR) Sassari Italy

**Keywords:** lncRNA, malaria, miRNA, ncRNA, *Plasmodium*

## Abstract

Malaria is one of the most severe infectious diseases affecting humans and it is caused by protozoan pathogens of the species *Plasmodium* (spp.). The malaria parasite *Plasmodium* is characterized by a complex, multistage life cycle that requires tight gene regulation which allows for host invasion and defense against host immune responses. Unfortunately, the mechanisms regulating gene expression during *Plasmodium* infection remain largely elusive, though several lines of evidence implicate a major involvement of non‐coding RNAs (ncRNAs). The ncRNAs have been found to play a key role in regulating transcriptional and post‐transcriptional events in a broad range of organisms including *Plasmodium*. In *Plasmodium* ncRNAs have been shown to regulate key events in the multistage life cycle and virulence ability. Here we review recent progress involving ncRNAs (microRNAs, long non‐coding RNAs, and circular RNAs) and their role as regulators of gene expression during *Plasmodium* infection in human hosts with focus on the possibility of using these molecules as biomarkers for monitoring disease status. We also discuss the surprising function of ncRNAs in mediating the complex interplay between parasite and human host and future perspectives of the field.

This article is categorized under:

RNA in Disease and Development > RNA in Disease

## Graphical abstract

The ncRNAs are emerging regulators of *Plasmodium* complex multistage life cycle and virulence gene expression. Also, ncRNAs are aberrantly produced in human host during infection constituting a promising biomarker. An improved understanding of ncRNAs related pathogenesis during malaria infection could greatly improve clinical management.

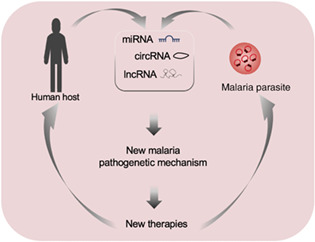

## 1 INTRODUCTION

Malaria is an infection caused by protozoan pathogens of the *Plasmodium* spp. It is one of the most prevalent infectious diseases worldwide and according to the World Malaria Report, an estimated 229,000 cases of malaria occurred in 2020 causing ∼65,000 deaths (World Malaria Report, 2020). Of more than 120 *Plasmodium* species only five are known to infect humans: *Plasmodium falciparum* (which is responsible for the most severe form of the disease), *Plasmodium vivax*, *Plasmodium malariae*, *Plasmodium ovale*, and *Plasmodium knowlesi* (Ashley et al., 2018; Miller et al., 1994). *P. falciparum* and *P. vivax* are the predominant species infecting humans worldwide (Howes et al., 2016).

**FIGURE 1:**
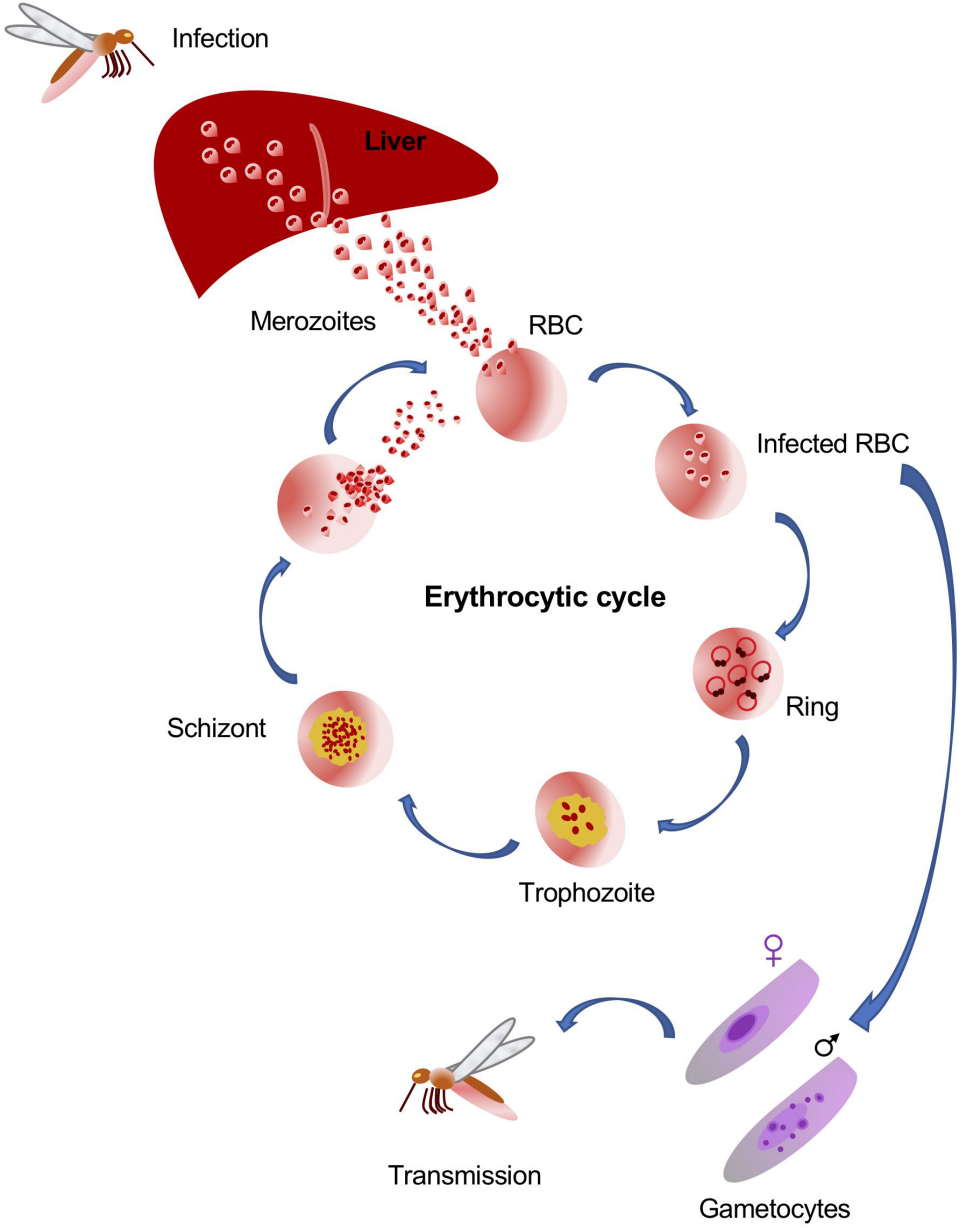
The *Plasmodium* life cycle

*Plasmodium* spp. have a complex life cycle that alternates between a mosquito vector of the Anopheles genus (the malaria vectors) and a vertebrate host (Pimenta et al., 2015). They are transmitted to humans during a blood meal, when *Plasmodium* sporozoites are injected by the bite of an infected mosquito female. Sporozoites, carried by the circulatory system to the liver, invade the hepatocytes. In the liver, the sporozoites mature into schizonts, which break and release merozoites into the blood stream (Figure 1).

Merozoites invade erythrocytes (at the beginning of the asexual blood stage) and undergo a trophic period (the trophozoite stage; Cowman et al., 2012; Phillips et al., 2017) at the end of which multiple rounds of nuclear division without cytokinesis lead to the formation of schizonts. The rupture of the schizonts in the blood stream releases merozoites, and the invasion of erythrocytes initiates another round of the blood‐stage replicative cycle. A portion of the merozoites differentiate and mature into male and female gametocytes, at this stage they can infect the mosquito host again during another blood meal (Figure 1; Bartoloni & Zammarchi, 2012).

The progression through the complex life cycle of *Plasmodium* requires tight regulation of gene expression, which occurs at both transcriptional and post‐transcriptional levels. Furthermore, as a survival adaptation to hostile variations present in host environments, regulating RNAs and proteins represents a fundamental step in the immune response of the protozoan organism (Hughes et al., 2010). The immunological response against the malaria parasite is complex, and the defense mechanisms are strongly affected by a multitude of antigens presented at different stages of the *Plasmodium* spp. life cycle. In humans, the immunological response to malaria antigens is mainly regulated through the cooperation of both the innate and adaptive immune systems with the immune attack higher during the erythrocytic stage (Hisaeda et al., 2005; Uchechukwu et al., 2017). Considerable evidence revealed that B cells, antibodies, T cells, cytokines, and their respective receptors, all play crucial roles in the recruitment and activation of different cell types of the immune system thus modulating the complex immunological response against malaria parasites (Deroost et al., 2016; Good et al., 1998). Indeed, the ability of the human organism to fight malaria infection relies on changes in gene expression that culminates in the activation of specific B‐cell and T‐cell subpopulations as well as cytokine production. Recently, for example, we investigated the effects of an increased production of soluble BAFF and downregulation of the RNA binding protein NF90 in modulating immune cell populations and cytokine production in the presence of malaria antigens (Idda et al., 2019; Lodde et al., 2020). In recent years, ncRNAs emerged as key modulators of gene expression by controlling heterogeneous events such as mRNA transcription, mRNA splicing, and translational efficiency (Pearson & Jones, 2016; Wright & Bruford, 2011).

The ncRNAs can be classified into structural (tRNA and rRNA) and regulatory ncRNAs. Regulatory ncRNAs, including microRNAs (miRNAs) and long non‐coding RNAs (lncRNAs), are expressed in specific cell types and in a time‐dependent manner to control particular outcomes (Z. Qu & Adelson, 2012; Dai et al., 2019). Interestingly, there is much evidence to suggest that ncRNAs could play a key role in numerous pathways implicated in the pathogenesis of infectious diseases including malaria (Drury et al., 2017; J. Chen et al., 2019; Shirahama et al., 2020; Tribolet et al., 2020). For example, Chakrabarti et al. (2007), Raabe et al. (2009), and Mourier et al. (2008) identified several types of ncRNAs, including miRNAs and lncRNAs with fundamental functions in the regulation of antigenic variation and virulence mechanism during *P. falciparum* infection. Recently additional evidence also suggests promising applications of ncRNAs in the prognosis and treatment of malaria infection (Rubio et al., 2016).

The recent major implications of ncRNAs in gene regulation prompted us to provide an overview of the latest studies analyzing the role of ncRNAs, miRNAs, lncRNAs, and circular RNAs (circRNAs), in the regulation of gene expression during *Plasmodium* infection in humans. Specifically, we investigate the possibility of using these molecules as biomarkers to monitor disease status as well as the surprising capacity of ncRNAs in mediating the interaction between human host and malaria parasite.

## 2 miRNAS IN MALARIA INFECTION

**FIGURE 2:**
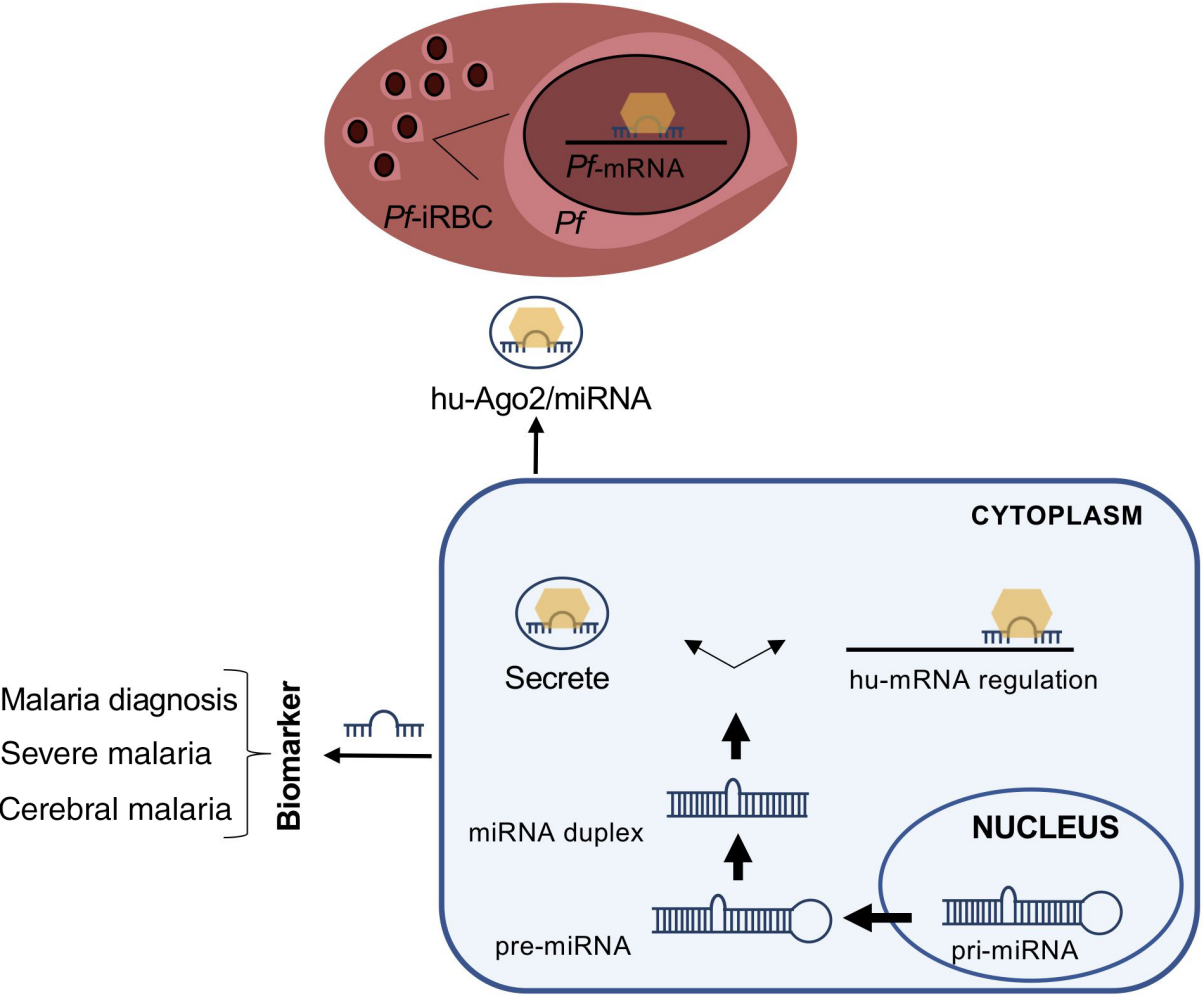
miRNAs biogenesis and function in response to malaria infection. miRNAs are transcribed by RNA PolII to produce pri‐miRNAs which are cleaved to generate pre‐miRNA that is transported to the cytoplasm to form mature miRNAs. After that, the single mature strand of miRNA is uploaded to the RISC complex, which contains Ago‐2 protein. During malaria infection miRNAs can regulate mRNA target or be secreted becoming possible biomarker or are imported by *P. falciparum* to modulate the expression of its own genes

The miRNAs are small ncRNAs (21–24 nts) that regulate gene expression in diverse biological processes. The miRNAs are initially transcribed as long primary (pri)microRNA transcripts and afterward, cleaved into 70–100 nucleotide long precursor miRNA (pre‐miRNA) (Gregory et al., 2004; Lee et al., 2003). The pre‐miRNA is exported from the nucleus to the cytoplasm by Exportin‐5/RanGTP. In the cytoplasm the endonuclease Dicer digests the pre‐miRNA into a 21–25 nucleotide miRNA duplex thus generating the mature miRNAs (Lund et al., 2004). The mature miRNAs are then selectively attached to a large complex of proteins termed the RNA‐induced silencing complex (RISC) which brings together the miRNA and its target on the mRNA through sequence‐specific interactions. The miRNA directs the RISC complex to target sites mainly located at the 3′‐untranslated regions (UTRs) leading to regulation of post‐transcriptional events such as RNA degradation and translational repression (Figure 2; Fabian & Sonenberg, 2012; Tang, 2005).

The miRNAs have been implicated in many cellular processes, such as cellular proliferation and differentiation, apoptosis, cytokine and chemokine production, inflammation, and immune response (Bueno & Malumbres, 2011; Chen et al., 2004; Munk et al., 2018; O'Connell et al., 2012; Rajman & Schratt, 2017; Salvi et al., 2019; Su et al., 2015).

Much evidence suggests that *Plasmodium* parasites are unable to produce miRNAs (Rathjen et al., 2006; Xue et al., 2008) and that both miRNA and human Argonaute 2 (hAgo2), a component of the RISC, are imported by *P. falciparum* to modulate the expression of its own genes. Indeed, in the parasite, hAgo2 exists as a complex with specific human miRNAs including let‐7a and miR15a which can, for example, target the *Plasmodium* gene Rad54 (Dandewad et al., 2019). On the other hand, malaria antigens can affect the production of organ‐specific host miRNAs, pointing toward the potential of these small molecules as biomarkers that can be used to reveal malaria associated immune responses and, in the worst cases, organ injury (Figure 2; Rubio et al., 2016). Thus, the content of miRNAs in the host cells and body fluids is influenced by host‐pathogen interactions (Hakimi & Cannella, 2011). For example, sequestration of *P. vivax* gametocytes in bone marrow has been associated with transcriptional changes of miRNAs involved in erythropoiesis, that in turn alter the expression of target mRNAs (Baro et al., 2017). A complete list of the miRNAs discussed in this manuscript is show in Table 1.

**TABLE 1:** miRNAs and lncRNAs in malaria

ncRNA name	Regulation	Biological sample	References
*miRNA*			
let‐7a	n.d.	RBCs	Dandewad et al. (2019)
miR‐15a	n.d.	RBCs	Dandewad et al. (2019)
miR‐16‐5p	Upregulated	WB	Dieng et al. (2020)
miR‐15a‐5p	Upregulated	WB	Dieng et al. (2020)
miR‐181c‐5p	Upregulated	WB	Dieng et al. (2020)
miR‐598‐3p	Upregulated	WB	Dieng et al. (2020)
miR‐146a	n.d.	WB	Van Loon et al. (2019)
miR‐451	Up and downregulated	Hbs, RBCs, plasma	Chamnanchanunt et al. (2015); Lamonte et al. (2012); Wang et al. (2017)
let‐7i	Upregulated	RBCs, mice brain	Chamnanchanunt et al. (2015); Lamonte et al. (2012); Wang et al. (2017)
miR‐221	Downregulated	Bone marrow	Baro et al. (2017)
miR‐222	Downregulated	Bone marrow	Baro et al. (2017)
miR‐24	Downregulated	Bone marrow	Baro et al. (2017)
miR‐191	Downregulated	Bone marrow	Baro et al., 2017
miR‐144	Upregulated	Bone marrow	Baro et al. (2017)
miR‐140	Upregulated	RBCs	Wang et al. (2017)
miR‐16	Downregulated	Plasma	Chamnanchanunt et al. (2015)
miR‐223	No change/UP	Plasma, RBCs	Chamnanchanunt et al. (2015); Lamonte et al. (2012)
miR‐226‐3p	No changes	Plasma	Chamnanchanunt et al. (2015)
miR‐7977	Upregulated	WB	Kaur et al. (2018)
miR‐28‐3p	Upregulated	WB	Kaur et al. (2018)
miR‐378‐5p	Upregulated	WB	Kaur et al. (2018)
miR‐194‐5p	Upregulated	WB	Kaur et al. (2018)
miR‐3667‐5p	Upregulated	WB	Kaur et al. (2018)
miR‐150‐5p	Upregulated	EV	Ketprasit et al. (2020)
miR‐15b‐5p	Upregulated	EV	Ketprasit et al. (2020)
Let‐7a‐5p	Upregulated	EV	Ketprasit et al. (2020)
miR‐3135b	Upregulated	WB	Li et al. (2018)
miR‐6780b‐5p	Upregulated	WB	Li et al. (2018)
miR‐1246	Upregulated	WB	Li et al. (2018)
miR‐6126	Upregulated	WB	Li et al. (2018)
miR‐3613‐5p	Upregulated	WB	Li et al. (2018)
miR‐4497	Upregulated	Plasma	Gupta et al. (2021)
let‐7i	Upregulated	Mouse brain	El‐Assaad et al. (2011)
miR‐150	Upregulated	Mouse brain	El‐Assaad et al. (2011)
miR‐27a	Upregulated	Mouse brain	El‐Assaad et al. (2011)
miR‐155	Upregulated	Mouse brain	Barker et al. (2017)
miR‐19a‐3p	Upregulated	Mouse brain	Martin‐Alonso et al. (2018)
miR‐19b‐3p	Upregulated	Mouse brain	Martin‐Alonso et al. (2018)
miR‐142‐3p	Upregulated	Mouse brain	Martin‐Alonso et al. (2018)
miR‐223‐3p	Upregulated	Mouse brain	Martin‐Alonso et al. (2018)
*lncRNA*			
TARE‐3	n.d.	*P.f*.	Sierra‐Miranda et al. (2012)
TARE‐6	n.d.	*P.f*.	Sierra‐Miranda et al. (2012)

Abbreviations: EV, extracellular vesicle; Hbs, hemoglobin; n.d., no define; P.f., Plasmodium falciparum; RBCs, red blood cells; WB, whole blood.

In the next sections we analyze two topics: (a) the modulation of miRNA expression in the human host induced by the presence of malaria antigens and (b) the ability of *Plasmodium falciparum* and *Plasmodium vivax* to import and embed the host miRNA machinery with the aim of modulating its own gene expression.

### 2.1 Modulation of human miRNA expression induced by *Plasmodium* infection

The clinical responses to infection and the development of effective responses to antimalarial drugs are marked by an interindividual variability also modulated by miRNA and post‐transcriptional events. To better understand these differences, Burel et al. used a controlled human infection model to study early immune events following primary infection of healthy human volunteers with blood‐stage *P. falciparum* malaria. They observed a dichotomous pattern of either high or low expression of a defined set of miRNAs that correlated with variations in parasite growth rate: 50% of individuals upregulated a set of miRNAs involved in immune responses (high‐miRNA responders), whereas the remaining volunteers downregulated the same miRNAs (low‐miRNA responders). The high‐miRNA responders had higher numbers of activated CD4+ T cells and developed a significantly enhanced antimalarial antibody response. Furthermore, prior to infection, in the whole blood of low‐miRNA responders, a set of 17 miRNAs was identified that differentiated them from high‐miRNA responders (Burel et al., 2017). A few years later, Dieng et al. performed an integrative genomic profiling and longitudinal study in a pediatric cohort from Burkina Faso. The authors reported a strong miRNA signature expression of a subset of miRNA during *P. falciparum* infection which correlate with infection and parasitemia. Over one‐third (127 out of 320) of the analyzed miRNAs, were significantly differentially expressed following *P. falciparum* infection in non‐infected children: integrative miRNA–mRNA analysis identified several infection‐responsive miRNAs including miR‐16‐5p, miR‐15a‐5p, and miR‐181c‐5p, promoting lymphocyte cell death. Furthermore, human miRNA cis‐eQTL analysis using whole‐genome sequencing data, identified 1376 genetic variants associated with the expression of 34 miRNAs. Specifically, they reported a protective effect of rs114136945 minor allele on parasitemia mediated by miR‐598‐3p expression (Dieng et al., 2020). Accordingly, a common miRNA‐146a polymorphism (rs2910164) increased the chances of *P. falciparum* malaria in pregnant African women (Van Loon et al., 2019). However, the same polymorphism was not associated with the odds ratio of malaria, irrespective of parasite species. These results also underline the importance of the genetic background relating to the complexity of clinical manifestations and the role of miRNAs during malaria infection (Van Loon et al., 2020).

An interesting aspect of malaria infection is the ability of red blood cells (RBCs) carrying the HbS variant in the hemoglobin gene (the molecular cause of sickle cell disease) to confer malaria resistance (Aidoo et al., 2002; Friedman, 1978). Indeed, La Monte et al. have investigated if miRNAs play a role in establishing this pattern. Remarkably, they found that, during the intraerythrocytic lifecycle of *P. falciparum*, a subset of erythrocyte miRNAs translocated into the parasite. In particular, the miRNAs miR‐451 and let‐7i are highly enriched in HbS erythrocytes and can regulate parasite growth. Surprisingly, they found that miR‐451 and let‐7i interact with crucial parasite mRNAs and induce translation inhibition through impaired ribosomal loading. Thus, modulation of miRNA expression in erythrocytes can influence the cell‐intrinsic resistance to malaria of sickle cell erythrocytes, representing a unique host defense strategy against complex eukaryotic pathogens (Lamonte et al., 2012).

To gain insights into *P. vivax* infection during the bone marrow phase, Baro et al. performed a morphological and molecular study on cells expressing CD71, a marker for bone marrow erythroid precursors, (Marsee et al., 2010) from bone marrow aspirated from a man diagnosed with *P. vivax* infection, before and after treatment (Baro et al., 2017). To identify possible bone marrow transcriptional changes related to erythropoiesis during infection, the expression profiles of small RNAs both during the acute attack and at convalescence were determined. Analysis of miRNAs related to erythropoiesis revealed a distinct series of differentially expressed miRNAs during *P. vivax* infection. For example, miR‐221/222, miR‐24, and miR‐191, which are normally downregulated during erythroid maturation, were decreased during *P. vivax* infection compared with convalescence. In contrast, miR‐144, which is upregulated during erythropoiesis, was found to be increased. These results indicate an altered miRNA profile in bone marrow erythropoiesis pathway during the acute *P. vivax* infection in the analyzed patient (Baro et al., 2017).

### 2.2 Extracellular vesicle‐derived miRNAs

Extracellular vesicles (EVs) are membranous cell‐derived vesicles originating from the endosomal system (exosomes) or by the outward budding and fission of the plasma membrane into the extracellular spaces (microvesicles) (Raposo & Stoorvogel, 2013). EVs (exosomes and microvesicles) transport proteins, nucleic acids (including ncRNAs), lipids, and so on from the host cells (Figure 2) (Zhang et al., 2019).

EVs have been extensively studied in malaria (Tsamesidis et al., 2020). Studies of circulating EVs from various cellular sources during *Plasmodium* spp. infection demonstrated an upregulation in EV secretion, thus demonstrating a key role of EVs in disease pathogenesis and prognosis. For example, RBC‐derived EVs concentrations in patients infected with either *P. vivax*, *P. malariae*, or *P. falciparum* are higher in patients affected by *P. falciparum* severe malaria (SM) (Pankoui Mfonkeu et al., 2010). Furthermore, it has been demonstrated that the EVs released during malaria infections generate a proinflammatory environment contributing to both SM and cerebral malaria (CM) onset, while genetic or pharmacological blockage of EV production reduces the development of CM in a mouse model (Babatunde et al., 2020; Cohen et al., 2018; Combes et al., 2005; Couper et al., 2010). Also, several studies reported that miRNAs can be transferred from one species to another through EVs, inducing species‐to‐species signaling, even in a cross‐kingdom manner. In general, these vescicles play important roles for intercellular communication and could potentially serve as biomarkers for different aspects of a specific disease (Tkach & Théry, 2016).

In line with these general considerations, in 2016 Mantel et al. demonstrated that inflammatory responses, during *Plasmodium* infection, are triggered in part by bioactive parasite products including infected RBC‐derived EVs. EVs contain functional miRNA–Argonaute 2 complexes that are derived from the host RBC. Moreover, they demonstrated that EVs are efficiently internalized by endothelial cells, and that the miRNA–Argonaute 2 complexes modulate target gene expression and barrier properties thus providing a mechanistic link between EVs and vascular dysfunction during malaria infection (Mantel et al., 2016).

Recent studies have also reported significant production of extracellular vesicles (microparticles, MPs) in the blood circulation of malaria patients, with RBCs being the major source of EV production. Wang et al. isolated the MPs from a culture medium of normal RBCs and malaria parasite‐infected RBCs (iRBCs), compared their quantity and origins and profiled miRNAs by RNA seq analysis. They observed a larger production of MPs in the culture media of iRBCs as compared with RBCs. Further investigation indicated that, in these MPs, hAgo2 associated with hundreds of miRNAs. These hAgo2–miRNA complexes were transferred into the parasites, and the expression of an essential malaria antigen PfEMP1, was downregulated by miR‐451/140. This report revealed, for the first time, that the malaria parasite can use human post‐transcriptional elements and mechanisms to modulate its own gene expression and underline the possibility of using miRNAs as potential drugs to treat malaria patients (Wang et al., 2017). Along the same line, Babatunde et al. isolated EVs from cultured iRBCs to study the content of regulatory RNAs. They found that miRNAs and tRNA‐derived fragments are the most abundant human RNAs. They also identified approximately 120 plasmodial RNAs, including mRNAs coding for exported proteins and proteins involved in drug resistance, as well as ncRNAs. These data demonstrated that iRBC‐EVs carry small regulatory RNAs and suggest their use as biomarkers for disease diagnosis and progression (Babatunde et al., 2020).

### 2.3 Extracellular miRNAs as biomarkers for malaria

Extracellular miRNAs, including plasma miRNAs, are highly stable (Reid et al., 2011) and the levels of some plasma miRNAs change as an effect of infectious diseases and organ damage (Chen et al., 2008; Mitchell et al., 2008) Thus, plasma miRNAs can be considered as possible non‐invasive biomarkers (Figure 2). One of the first studies on this topic was published by Chamnanchanun et al. (2015). To identify new biomarkers for malaria infection, they analyzed plasma miRNAs from 19 malaria patients and 19 normal subjects, using reverse transcription‐based quantitative polymerase chain reaction (RT‐qPCR). They showed that the plasma levels of miR‐451 and miR‐16 were downregulated in patients with *P. vivax* infection, and suggested a correlation with the severity of parasitemia (Chamnanchanunt et al., 2015). By contrast, the levels of other abundant miRNAs in plasma (miR‐223, miR‐226‐3p) did not change significantly in malaria patients. More recently, Kaur et al., investigated the expression of miRNAs from total RNA extracted from whole blood samples of healthy controls, which were negative for *P. vivax*, and *P. vivax* complicated and uncomplicated malaria using Affymetrix miRNA array. The authors identified a total of 276 miRNAs differentially expressed, out of which five miRNAs (miR‐7977, miR‐28‐3p, miR‐378‐5p, miR‐194‐5p, and miR‐3667‐5p) were found to be significantly upregulated in complicated *P. vivax* malaria patients. MiR‐7977, which was the most upregulated in complicated *P. vivax*, may have a role in the infection pathology, probably through regulation of the TGFβ signaling pathway. It was also postulated that miR‐7977 may be used as a potential biomarker to distinguish between complicated versus uncomplicated *P. vivax* infection (Kaur et al., 2018).

More recently, Ketprasit et al. analyzed miRNA expression in EVs purified from the plasma of Thai *P. vivax*‐infected patients, *P. falciparum*‐infected patients and uninfected individuals. In their experimental conditions the relative expression of miR‐150‐5p and miR‐15b‐5p was higher in *P. vivax*‐infected patients as compared with uninfected individuals, while let‐7a‐5p was upregulated in both *P. vivax*‐infected and *P. falciparum*‐infected patients. Using bioinformatic tools they also observed that these miRNAs may regulate key genes involved in the malaria pathway such as the adherent junctions and the transforming growth factor‐β pathways. The identified miRNAs could potentially be used as disease biomarkers but further investigation is required to validate sensitivity and specificity (Ketprasit et al., 2020).

Malaria remains the most significant imported parasitic infection in North America and Europe (Mali et al., 2012; Odolini et al., 2012), of which *P. falciparum* is both the most common and the most severe. Correlating altered miRNA expression during the blood stage of imported malaria is required to better understand the in vivo biological and molecular processes involved in the response to *P. falciparum* infection and to find new biomarkers and diagnosis tools. To this end, Li et al. used a parallel microarray‐based approach to obtain an integrated view of how the host miRNAs expression profile changes in response to *P. falciparum* infection. Whole blood from six subjects with adult imported *P*. *falciparum* malaria (AIFM) was compared with six normal subjects. They identified five upregulated miRNAs (miR‐3135b, miR‐6780b‐5p, miR‐1246, miR‐6126, and miR‐3613‐5p), which can act as potential blood biomarkers of immunopathological status and prediction of early host responses, disease prognosis, and severe outcomes in AIFM (Li et al., 2018).

### 2.4 Severe malaria and cerebral malaria

SM and CM, two key complications of malaria infection, are injurious health problems in endemic areas, especially when considering the widespread issue of malarial drug resistance and the lack of an effective vaccine (Postels & Birbeck, 2013; WHO, 2014).

#### 2.4.1 Severe malaria

SM occurs when infections are complicated by vital organ dysfunctions or aberrant metabolism (WHO, 2014). During infection, *P. falciparum* infected erythrocytes can be sequestrated in vital organs which leads to inflammation and possible organ impairment. These events correlate with a rapid release of miRNAs into the host fluids that can be detected as promising biomarkers for the prognosis of SM. Recently, using next‐generation sequencing, Gupta et al. evaluated the differential expression of miRNAs in SM and in uncomplicated malaria (UM) in children living in Mozambique. They identified six miRNAs associated with in vitro *P. falciparum* cytoadhesion, severity, and *P. falciparum* biomass. Among them, levels of miR‐4497 were higher in the plasma of children affected by SM as compared with UM and correlated with *P. falciparum* biomass. These findings suggest that different physio‐pathological processes in SM and UM lead to differential expression of miRNAs suggesting a way for assessing their prognostic value (Gupta et al., 2021).

#### 2.4.2 Cerebral malaria

CM is the most severe neurological complication of malaria, whose hallmark is impaired consciousness, with coma being the most severe manifestation (Idro et al., 2010). CM onset is a complex event involving multiple alterations, including aberrant levels of proinflammatory cytokine interferon‐γ (IFN‐γ) and tumor necrosis factor alpha (TNF‐α), aggregation of inflammatory cells in the cerebral blood vessels, tissue sequestration of infected RBCs, and apoptosis. As stated above, a relevant function of miRNAs in malaria pathogenesis has been identified, and a contribution in CM onset recently demonstrated. Unfortunately, the study of miRNA in human CM is still on its infancy, thus, we decided to focus on mouse studies for this section.

To deeply understand the role of miRNA in the immune response to *Plasmodium* during CM El‐Assaad et al. used brain tissue of *Plasmodium* infected mice: they have shown a significant upregulation in the expression of let‐7i, miR‐150, and miR‐27a suggesting their critical involvement in the severity of CM (El‐Assaad et al., 2011). The family of let‐7 miRNAs is described as controlling cellular proliferation and the innate immune response. miR‐150 is highly expressed in monocytes and has a role in cell proliferation and apoptosis, while miR‐27a is involved in apoptosis induction, regulation of T cell proliferation, and activation of the NF‐κB signaling pathway (Chhabra et al., 2009; O'Hara et al., 2010; Tourneur & Chiocchia, 2010). The upregulation of these miRNAs and modulation of their potential targets during malaria infection may be crucial for CM development.

Ex vivo endothelial microvessel and mouse models have been used by Barker et al, to describe the potential role of miR‐155 in CM. miR‐155 is a negative regulator of endothelial and blood–brain barrier (BBB) integrity during SM. miR‐155 targets ANXA2 mRNA that binds VE‐cadherin which is required for endothelial barrier function. Interestingly, in this study, deletion of *miR‐155* resulted in decreased endothelial activation, increased BBB integrity, and increased T cell function, improving clinical outcomes during CM (Barker et al., 2017). These results point to new therapeutic strategies inhibiting miR‐155; further investigations are need to deeply investigate this possibility.

In another mouse model of CM, the expression of miRNAs was studied following infection with *Plasmodium berghei* (causing CM) or *Plasmodium yoelii* (causing severe but non‐cerebral malaria [NCM]). Using microarray analysis, miRNA expression was analyzed in the brains of non‐infected (NI), NCM, and CM mice. Four dysregulated miRNAs were identified and validated in CM mice as compared with NCM, miR‐19a‐3p, miR‐19b‐3p, miR‐142‐3p, and miR‐223‐3p. These miRNAs are involved in key pathways implicated in CM onset, including the TGF‐β and endocytosis pathways, vitally involved in the neurological syndrome. This data implies that, at least in the mouse model, miRNAs may play a regulatory role in CM pathogenesis (Martin‐Alonso et al., 2018).

All together, these results demonstrated the relevance of miRNAs for SM and CM onset, diagnosis, and prognosis while providing further incentive to deeply study the potential role of miRNAs in human CM.

## 3 lncRNAS AND circRNAS IN MALARIA INFECTION

The lncRNAs and circRNAs are classes of ncRNAs longer than 200 nts, usually characterized by the absence of protein‐coding capabilities. Both lncRNAs and circRNAs have a significant role in a variety of key biological processes, modulating gene expression at different levels including transcription, post‐transcription, RNA turnover and translation, and protein translation [89]–[93] (Salzman, 2016; Yu & Kuo, 2019). Besides miRNAs, lncRNAs and circRNAs are also produced by *Plasmodium* protozoa and like miRNAs, they are also involved in the regulation of key biological processes during malaria infection (Broadbent et al., 2015; Yiran Li et al., 2020).

### 3.1 lncRNAs

**FIGURE 3:**
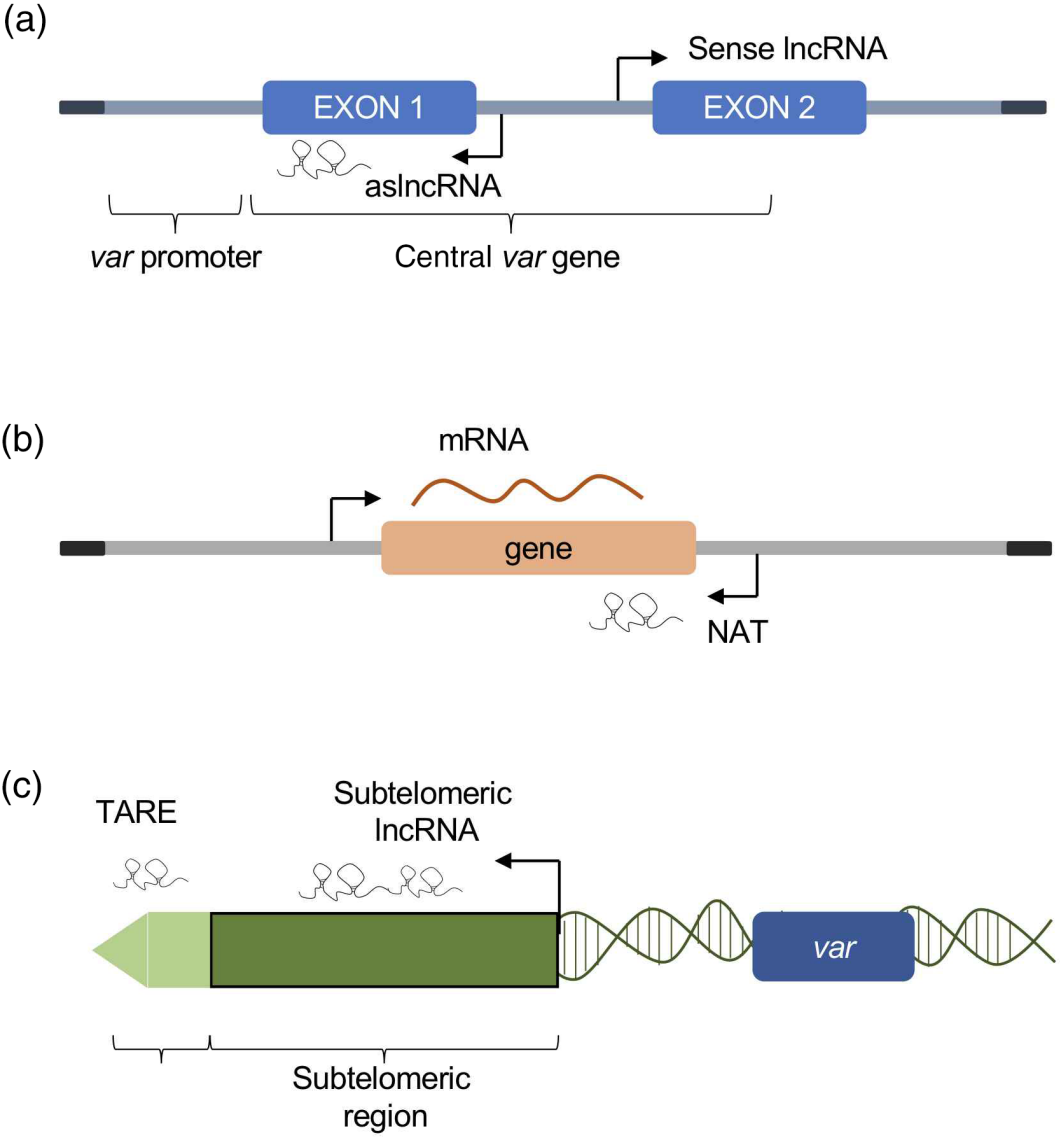
Schematic representation of gene structures and lncRNAs generation in *P. falciparum*. (a) *var* gene and *aslncRNAs*. (b) *NATs lncRNA* from general gene. (c) Sub‐telomeric and telomeric lncRNAs

The lncRNAs, transcribed by Pol II, are characterized by the presence of 5′ caps and poly(A) tails, and undergo splice maturation similarly to mRNAs (Erdmann et al., 2001; Fernandes et al., 2019). According to the genomic region of transcription, they can be classified as intergenic, intronic, sense lncRNAs, transcribed from the sense strand of protein‐coding genes, and natural antisense lncRNAs (NATs) that are transcribed from the antisense strand of protein‐coding genes (Figure 3; Ma et al., 2013).

Several lncRNAs have been functionally associated with human diseases, including infectious diseases such as malaria (Fang & Fullwood, 2016; Ginn et al., 2020; Lodde, Murgia, et al., 2020; Shirahama et al., 2020; Wapinski & Chang, 2011). As an example, Liao et al. identified 164 novel *P. falciparum* lncRNAs, and predicted functions for 69 of them. The main functions of these lncRNAs involved metabolic and catabolic processes, cellular organization, as well as regulation of biological processes. Several of the identified lncRNAs cooperate with proteins that are important in the host–parasite interaction, such as the MSP family, RH5, and CLAG3 and might be crucial in the invasion of *P. falciparum* into erythrocytes (Liao et al., 2014). A few studies, summarized here, are considering the role of lncRNAs during malaria infection and their contribution to the expression and regulation of virulence genes as well as human response. A complete list of the lncRNAs analyzed in this manuscript is shown in Table 1.

#### 3.1.1 Pf‐var‐aslncRNAs

The virulence of malaria parasite is mainly due to the ability of *Plasmodium* to evade human immunity through antigenic variation. The antigenic variation depends on the ability of this protozoa to alternate between expression of the variable antigens, encoded by members of a multicopy gene family named *var*. Tight regulation of the *var* genes expression, ensures that only a single *var* gene is expressed at a time while the rest of the family is maintained through transcriptional silence (Claessens et al., 2014; Scherf et al., 1998). Recently, intronic *var* gene antisense long non‐coding RNAs (*var‐aslncRNAs*) have been identified as possible candidates in the regulation of the *var* genes. Indeed, the transcription of the *var‐aslncRNAs* was correlated with the activation of a specific *var* gene (Amit‐Avraham et al., 2015; Jiang et al., 2013). To further study this regulatory mechanism in depth, Jing et al. synthesized in vitro an exogenous artificial *var*‐*aslncRNA* using T7 RNA polymerase and demonstrated that they can specifically activate the homologous *var* gene (Figure 3a; Jing et al., 2018). These results demonstrated that *var*‐*aslncRNAs* are responsible for *var* gene transcriptional regulation and thus for antigenic variation.

#### 3.1.2 *Pf*‐natural antisense transcript

Many studies reported the presence of natural antisense transcripts (NATs) in *Plasmodium* parasite and investigated the role of NATs in the regulation of gene expression (Militello et al., 2005). Using serial analysis of gene expression (SAGE) in erythrocytic stages, Patankar et al. show that *Plasmodium* parasites express antisense RNAs during multiple stages throughout the developmental cycle (Patankar et al., 2001). Furthermore, the presence of NATs in gametocyte and ookinete genes suggests that these antisense RNAs may play a critical role in gene expression regulation and parasite development (López‐Barragán et al., 2011). Additional evidence supporting the presence of NATs has been subsequently reported from different groups (López‐Barragán et al., 2011; Siegel et al., 2014; Sorber et al., 2011). Subudhi et al., for example, investigated the presence of NATs using a custom designed strand specific whole genome microarray in *P. falciparum* which was directly isolated from patients with uncomplicated and complicated malaria. They identified a total of 545 unique NATs with the majority positively correlating with the expression pattern of the sense transcript. The identified NATs mapped to a broad range of biochemical/metabolic pathways, including stress related pathways (Figure 3b). They also observed that the expression pattern of NATs change between the two different clinical conditions analyzed, uncomplicated, and complicated malaria (Subudhi et al., 2014).

#### 3.1.3 Pf‐lncRNA‐TAREs

The lncRNAs interact with RNA binding proteins and chromatin remodeling complexes regulating their functions (Mercer et al., 2009). To further analyze this aspect, Broadbent et al. investigated the role of lncRNAs of the *P. falciparum* strain 3D7 genome using a high‐resolution DNA tiling array. They identified a family of 22 telomere‐associated lncRNAs, termed *lncRNA‐TARE* (telomere‐associated repeat elements). The *lncRNA‐TARE* loci are expressed after parasite DNA replication and are thought to play an important role in *P. falciparum* telomere maintenance and virulence gene regulation (Broadbent et al., 2011). Moreover, Sierra‐Miranda et al. showed that TAREs are transcribed as lncRNAs during the schizont stage (Figure 3c). In particular, they discovered that two *lncRNA‐TAREs*, *TARE‐3*, and *TARE‐6*, form several nuclear foci during the schizont stage, while in the ring stage they are processed into shorter and more stable ncRNAs and are located in a single perinuclear compartment. The biological function of this compartment remains unclear, possible the lncRNAs may recruit specific nuclear proteins and the interactions of these lncRNAs with proteins may reveal novel modes of gene regulation and nuclear function in *P. falciparum* (Sierra‐Miranda et al., 2012).

Overall, these studies revealed that lncRNAs produced by *P. falciparum* may have a crucial role in the regulation of genes involved in infection and virulence modulating relevant steps at both the transcriptional and post‐transcriptional levels.

### 3.2 circRNAs

The circRNAs are covalently linked single‐stranded RNAs without a 5′ cap or 3′ tail (S. Qu et al., 2015). They are predominantly found in the cytoplasm and in extracellular fluids (Y. Li et al., 2015). Based on their structure, circRNAs can be classified as exonic circular RNAs (ecircRNAs), that can be divided into single‐exon circRNAs or multiexon circRNAs, circular intronic RNAs (ciRNAs), and exon–intron circRNA (EIciRNAs). CircRNAs act as decoys for miRNAs and RBPs, modulating post‐transcriptional gene expression (Salzman, 2016; Yu & Kuo, 2019). The existence of mammalian circRNAs was first reported in 1979 by Hsu et al. and in 2014 Wang et al., for the first time, demonstrate the presence of circRNAs in *P. falciparum* (Coca‐Prados & Hsu, 1979; Wang et al., 2014). Following the approach used by Memczak et al. (2013), Broadbent et al. identified 1381 putative *P. falciparum* circRNAs. Only 72 of the 1381 circRNAs were predicted to be 200 bp or longer and 9 out of the 72 circRNAs were selected for further analysis. Using the recently described PACCMIT‐cds algorithm, they found that each of the nine circRNA candidates contained predicted human miRNA binding sites. They hypothesized that the *P. falciparum* transcripts had the capacity to form stable circular structures and the possibility to sponge human miRNAs to regulate their own gene expression (Broadbent et al., 2015). Further studies are needed to clearly understand how circRNAs regulate *Plasmodium* gene expression and affect infection and virulence capacity.

## 4 CONCLUSION

In *Plasmodium*, the progression through the complex life cycle as well as the survival against the human immune response are fundamental for protozoa fitness and tight gene regulation is required. Likewise, in humans, complex gene regulation orchestrates a fine modulation of genes essential to counteract the infection. In recent years, with the increased knowledge in the field of ncRNAs, several studies are considering their functions in modulating gene expression during malaria infection in both human and parasitic organisms. ncRNAs have an important role in the regulation of different processes, such us transcription, post‐transcriptional events, and maintenance of chromatin structure. Not unexpectedly, there is an increasing interest in understanding the level of involvement of ncRNAs during *P. falciparum* infection, especially in the modulation of virulence genes. Actually, *P. falciparum* ncRNAs with defined functions include, but are not limited to, human miRNAs able to modulate *P. falciparum* RNA, subtelomeric lncRNAs and virulence gene‐associated *var‐aslncRNAs*. In the next years, the improvement in high‐throughput sequencing approaches will expand this repertoire providing new directions for understanding infection pathogenesis and therapy. Furthermore, the adaptation of well‐known technologies, such as single‐cell RNA‐sequencing, is fundamental to uncover previously hidden transcriptional signatures characteristic of specific part of the *Plasmodium* life cycle as recently shown by Reid et al. (2018).

The interest of researchers for ncRNAs in gene expression modulation originates with the identification of miRNAs able to interact with the 3′ untranslated region of mRNAs and affecting protein synthesis. Subsequently, the discovery of lncRNAs and circRNAs without a clear ORF provided a new line of regulation. Hundreds of studies rapidly focused on the regulatory functions characterizing these molecules in signaling or metabolic pathways, including the infectious disease caused by *Plasmodium* spp. It is important to mention that the annotation of circRNAs and lncRNAs as non‐coding is controversial, indeed, new evidence has demonstrated that some can be translated into peptides with defined biological functions (Wu et al., 2020). Consequently, an exhaustive characterization of the *Plasmodium* ncRNAs will necessitate a parallel delineation of the parasite's translatome. To this end several approaches, adapted to malaria parasite, such as ribosome‐profiling and polysome‐profiling technique can be used (Jin & Xiao, 2018).

In cases of newly identified ncRNAs, it is fundamental the functional characterization by a selective and specific knockdown of a target *Plasmodium* transcripts. Primary approaches such as CRISPR/Cas and non‐canonical RNAi are under optimization to be high efficient in Plasmodium organisms (Hentzschel et al., 2020; Zhao et al., 2021). Using these approaches, the deletion of a specific *Pf‐var‐aslncRNAs* could be important in understanding their contribution to the mutually exclusive *var* gene expression. Moreover, new approaches to predict computationally the ncRNAs function have been developed and could be used for *Plasmodium* studies (Fukunaga et al., 2019; Noviello et al., 2020).

Identifying and characterizing parasite‐specific ncRNAs and their targets in hosts, as well as miRNAs, lncRNAs and circRNAs interfering with host pathology, are crucial for a better understanding of the pathophysiology of malaria infection at the molecular level. Furthermore we belive that the detection of new ncRNAs with highly specialized functions may be able to explain physiological and cellular processes providing innovative strategies for malaria treatment.

## CONFLICT OF INTEREST

The authors have declared no conflicts of interest for this article.

## AUTHOR CONTRIBUTIONS

**Valeria Lodde:** Conceptualization (equal); writing – original draft (equal); writing – review and editing (equal). **Matteo Floris:** Writing – review and editing (equal). **Maria Rosaria Muroni:** Writing – review and editing (equal). **Francesco Cucca:** Writing – review and editing (equal). **M. Laura Idda:** Conceptualization (equal); writing – original draft (equal); writing – review and editing (equal).

## RELATED WIREs ARTICLE

From cradle to grave: RNA biology in malaria parasites

## Data Availability

Data sharing is not applicable to this article as no new data were created or analyzed in this study.
